# Viruses Identified in Shrews (*Soricidae*) and Their Biomedical Significance

**DOI:** 10.3390/v16091441

**Published:** 2024-09-10

**Authors:** Huan-Yu Gong, Rui-Xu Chen, Su-Mei Tan, Xiu Wang, Ji-Ming Chen, Yuan-Long Zhang, Ming Liao

**Affiliations:** 1 School of Animal Science and Technology, Foshan University, Foshan 528225, China; huanyugong@outlook.com (H.-Y.G.); raycechan@163.com (R.-X.C.); sumeitan321@outlook.com (S.-M.T.); 15922471545@163.com (X.W.); 2 Guangdong Center for Animal Disease Prevention and Control, Guangzhou 510230, China; 3 College of Animal Science and Technology, Zhongkai University of Agriculture and Engineering, Guangzhou 510230, China

**Keywords:** shrew, virus, diversity, public health, epidemiology, risk

## Abstract

Shrews (*Soricidae*) are common small wild mammals. Some species of shrews, such as Asian house shrews (*Suncus murinus*), have a significant overlap in their habitats with humans and domestic animals. Currently, over 190 species of viruses in 32 families, including *Adenoviridae*, *Arenaviridae*, *Arteriviridae*, *Astroviridae*, *Anelloviridae*, *Bornaviridae*, *Caliciviridae*, *Chuviridae*, *Coronaviridae*, *Filoviridae*, *Flaviviridae*, *Hantaviridae*, *Hepadnaviridae*, *Hepeviridae*, *Nairoviridae*, *Nodaviridae*, *Orthoherpesviridae*, *Orthomyxoviridae*, *Paramyxoviridae*, *Parvoviridae*, *Phenuiviridae*, *Picobirnaviridae*, *Picornaviridae*, *Polyomaviridae*, *Poxviridae*, *Rhabdoviridae*, *Sedoreoviridae*, *Spinareoviridae*, and three unclassified families, have been identified in shrews. Diverse shrew viruses, such as Borna disease virus 1, Langya virus, and severe fever with thrombocytopenia syndrome virus, cause diseases in humans and/or domestic animals, posing significant threats to public health and animal health. This review compiled fundamental information about shrews and provided a comprehensive summary of the viruses that have been detected in shrews, with the aim of facilitating a deep understanding of shrews and the diversity, epidemiology, and risks of their viruses.

## 1. Introduction of Shrews

Shrews, which are usually termed long-nosed mice, can be found in a variety of habitats, including forests, grasslands, deserts, and wetlands. Some species, such as American water shrews (*Sorex palustris*), are aquatic and can be found in or near water.

Asian house shrews (*Suncus murinus*) are the largest shrews, attaining a length of approximately 15 cm and a weight of around 100 g, while Etruscan shrews (*Suncus etruscus*) could represent the smallest extant terrestrial mammals, measuring a mere 3.5 cm in length and weighing approximately 1.8 g [1].

Shrews, hedgehogs, and moles share the same order, *Eulipotyphla.* They belong to the families *Soricidae*, *Erinaceidae*, and *Talpidae*, respectively. *Eulipotyphla* encompasses 593 species, second only to *Rodentia* (2398 species) and *Chiroptera* (1449 species) in mammals [2]. These three orders comprise about 9.5%, 38.5%, and 23.2% of all mammalian species, respectively.

Of the known 163 mammalian families, *Soricidae* encompasses 487 species, only less than *Muridae* (775 species), *Cricetidae* (705 species), and *Vespertilionidae* (520). The 488 species of *Soricidae* belong to three subfamilies and 28 genera [2]. The three subfamilies are *Crocidurinae* (white-toothed shrews), *Soricinae* (red-toothed shrews), and *Myosoricinae* (African shrews). These three subfamilies comprise 10 genera with 267 species, 15 genera with 195 species, and three genera with 25 species, respectively. The genus *Crocidura* is the largest genus in *Crocidurinae*, covering 222 species. The genus *Sorex* is the largest genus in *Soricinae*, covering 86 species [2].

Notably, tree shrews in the order *Scandentia*, otter shrews in the order *Afrosoricida*, elephant shrews in the order *Macroscelidea*, and marsupial shrews in the order *Dasyuromorphia* are not shrews, because they do not fall within the order *Eulipotyphla* [3]. West Indies shrews were in the order *Eulipotyphla*, but they were in the family *Nesophontidae* and have been extinct.

Shrews are distributed across major tropical and temperate landmasses on all continents except Australia and Antarctica (Table 1). Oceania is devoid of shrews, except on two islands of the Mariana Archipelago (Guam and Tinian), where *Suncus murinus* was introduced following World War II [4]. In South America, shrews are confined to the northern Andes region and were introduced during the Great American Interchange.

Shrews have a lifespan of 12 to 30 months, and they exhibit a relatively high metabolic rate, higher than some mammals of similar body sizes. Consequently, shrews have a relatively large food intake. Shrews do not hibernate but can enter a torpid state. During the winter, many shrews undergo significant changes in morphology, with body weight decreasing by 30% to 50% and both skeletal and organ sizes noticeably shrinking [5]. Shrews are mostly found in cool and humid environments, with many being terrestrial, while some are semi-aquatic or burrowing. They may be active during both day and night or primarily nocturnal. Shrews are typically solitary creatures, coming together only for mating purposes.

Female shrews can give birth to as many as 10 litters each year. In tropical regions, they can reproduce year-round, while in temperate regions, they cease reproduction during the winter. The gestation period for shrews ranges from 17 to 32 days. Female shrews can become pregnant again within one to two days after giving birth and can lactate and nurse their offspring during pregnancy [6].

Certain shrew species, such as American or southern short-tailed shrews (*Blarina carolinensis*), northern short-tailed shrews (*Blarina brevicauda*), and long-tailed shrews or rock shrews (*Sorex dispar*) in North America, and Asiatic short-tailed shrews (*Blarinella quadraticauda*) in China, secrete venom [7], which contains various compounds. The venom of American short-tailed shrews can kill 200 mice when administered intravenously [8]. In addition, similar to bats and toothed whales, two genera, namely *Sorex* (long-tailed shrews) and *Blarina* (short-tailed shrews), possess echolocation abilities [9]. These two genera include Eurasian water shrews (*Neomys fodiens*), northern short-tailed shrews (*Blarina brevicauda*), American water shrews, and some others [10].

**Figure 1:**
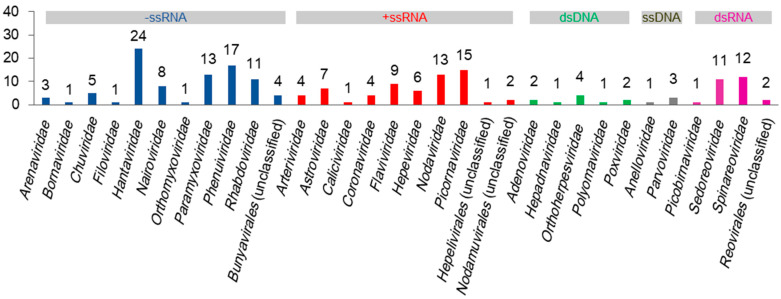
The numbers of virus species in 32 virus families identified in shrews.

Diverse viruses in shrews have been identified, and many viruses in shrews have yet to be identified [11]. Currently, as detailed below and summarized in Figure 1, at least 190 species of viruses in 32 families have been identified in shrews, excluding plant viruses or invertebrate viruses that might be detected in shrews due to their existence in the food or surroundings of shrews. These viruses can be classified into negative-sense single-stranded RNA (−ssRNA), positive-sense single-stranded RNA (+ssRNA), double-stranded DNA (dsDNA), single-stranded DNA (ssDNA), and double-stranded RNA (dsRNA) viruses (Figure 1). The relevant sequences of these viruses are provided in the supplementary file (Table S1).

## 2. −ssRNA Viruses Detected in Shrews

Table 2 shows that 88 species of −ssRNA viruses in shrews have been detected.

### 2.1. Arenavirid Viruses Detected in Shrews

The family *Arenaviridae* currently comprises five genera and 69 species, which infect mammals, reptiles, and fish [51,52]. Their genomes consist of two or three single-stranded RNA molecules, some of which encode two proteins in non-overlapping ORFs of opposite polarities. Some viruses in this family, such as lymphocytic choriomeningitis virus and Lassa virus, can cause diseases in humans.

Three species in the genus *Mammarenavirus* (*M. wenzhouense*, *M. choriomeningitidis*, and an unclassified species) of this family were identified in shrews. *M. wenzhouense* and an unclassified species of *Suncus murinus* in China were reported in recent years [12,13,15], and *M. choriomeningitidis* in goliath shrews (*Crocidura goliath*) in Gabon was reported in 2021 [14]. Of them, *M. wenzhouense* (Wēnzhōu virus) can infect humans, causing influenza-like symptoms [12,13]. *M. choriomeningitidis* (lymphocytic choriomeningitis virus) can infect humans and primates, causing infection in the central nervous system. This virus can also cause other illnesses, such as conjunctivitis, hepatitis, pneumonia, meningitis, and sepsis [51,52]. Shrews could be accidental hosts or a minor reservoir of these two virus species, which mainly circulate in rodents [51,52].

### 2.2. Bornavirid Viruses Detected in Shrews

The family *Bornaviridae* currently comprises the genera *Carbovirus*, *Cultervirus*, and *Orthobornavirus*, which infect mammals, birds, reptiles, and fish [53]. Several viruses in this family, such as *Orthobornavirus bornaense* (Borna disease virus 1, BDV-1) and variegated squirrel bornavirus 1, can cause diseases in humans or domestic animals.

BDV-1 in the genus *Orthobornavirus* of this family causes severe T-cell-mediated meningoencephalitis in horses, sheep, and other animals, especially in central Europe. Recent studies have identified white-toothed shrews (*Crocidura leucodon*) as the natural host and reservoir of BDV-1 [54,55]. This virus was detected in white-toothed shrews (*Crocidura russula*) in Germany in recent years (Table 2). BDV-1 can persist in the shrews for long periods without noticeable clinical signs of illness and can be shed through shrew saliva, urine, skin swabs, tears, and feces.

### 2.3. Chuvirid Viruses Detected in Shrews

The family *Chuviridae* currently comprises 16 genera and 43 species, which infect arachnids, barnacles, crustaceans, insects, fish, and reptiles in Africa, Asia, Australia, Europe, North America, and South America [56].

Five unclassified species of *Chuviridae* with unknown pathogenicity were identified in Asian grey shrews (*Crocidura attenuata*), Smith’s shrews (*Chodsigoa smithii*), and Ussuri white-toothed shrews *(Crocidura lasiura*) in China in recent years [11,17].

### 2.4. Filovirid Viruses Detected in Shrews

The family *Filoviridae* currently comprises nine genera and 16 species, which infect mammals, reptiles, and fish [57]. Several viruses in this family, such as Ebola virus and Marburg virus, can cause severe diseases in humans and animals.

Ebola virus in the genus *Orthoebolavirus* of this family is highly pathogenic to humans and has been detected in greater forest shrews (*Sylvisorex ollula*) in the Central African Republic, but shrews could be intermediate or incidental hosts of this virus [58].

### 2.5. Hantavirid Viruses Detected in Shrews

The family *Hantaviridae* currently comprises four subfamilies, eight genera, and 53 species, which infect mammals, birds, reptiles, and fish [59]. Multiple viruses in this family, such as Hantaan virus and Seoul virus, can cause diseases in humans.

One species in the genus *Mobatvirus* of this family, *M. lenaense* (Lena virus), was discovered in Laxmann’s shrews (*Sorex caecutiens*) and flat-skulled shrews (*Sorex roboratus*) captured between 2018 and 2019 in Russia [19].

Diverse species in the genus *Orthohantavirus* of this family, such as *O*. *artybashense*, *O*. *asikkalaense*, *O*. *boweense*, *O*. *caobangense*, *O*. *jejuense*, *O*. *kenkemeense*, *O*. *seewisense*, *O*. *seoulense*, and several unclassified species, were detected in multiple countries (Table 2). Of them, *O*. *seoulense* (Seoul virus) is highly pathogenic to humans with a reservoir of rodents. This virus was discovered in *Crocidura lasiura* captured in 2014 in China [26]. It remains unknown whether shrews are the natural hosts or a reservoir of the virus.

In the genus *Orthohantavirus*, *O*. *seewisense* (Seewis virus) was first detected in Eurasian common shrews *(Sorex araneus*) captured in 2006 in Switzerland [20]. This virus is widely distributed throughout the geographic ranges of its soricid hosts, including *Sorex araneus*, tundra shrews (*Sorex tundrensis*), Siberian large-toothed shrews (*Sorex daphaenodon*), and Mediterranean water shrews (*Neomys anomalus*). In addition, some genetic variants of this virus, termed Artybash virus and Amga virus, were detected in *Sorex caecutiens* [27]. *O*. *caobangense* (Cao Bằng virus) was first detected in the lung tissue of Chinese mole shrews *(Anourosorex squamipes*) captured in 2006 in Vietnam [60]. *O*. *asikkalaense* (Asikkala virus) was first discovered in Eurasian pygmy shrews (*Sorex minutus*) captured in Finland [21]. Subsequently, this virus was identified in shrews in the Czech Republic and Germany. *O*. *boweense* (Bowé virus) was discovered in the muscle tissue of Doucet’s musk shrews (*Crocidura douceti*) captured in 2012 in southwestern Guinea [22]. *O*. *jejuense* (Jeju virus) was first discovered in Asian lesser white-toothed shrews (*Crocidura shantungensis*) captured between 2007 and 2010 in South Korea [24]. *O*. *kenkemeense* (Kenkeme virus) was first discovered in *Sorex roboratus* captured in 2006 in Russia [25].

In the genus *Orthohantavirus*, the unclassified Altai virus was first detected in the tissue of *Sorex araneus* in the Altai Republic of Russia in 2007 [29]. The unclassified Ash River virus was discovered in masked shrews (*Sorex cinereus*) captured in 1983 and 1994 in Minnesota. The unclassified Jemez Springs virus was discovered in *Sorex monticolus* captured in 1994 in Colorado and between 1996 and 2000 in New Mexico [31]. The unclassified Artybash virus was first detected in the lung of *Sorex caecutiens* captured between 2006 and 2014 in eastern Siberia, Russia, and Hokkaido, Japan [20]. The unclassified Azagny virus was first discovered in West African pygmy shrews (*Crocidura obscurior*) captured in 2009 in Côte d’Ivoire [34]. The unclassified Boginia virus was first reported in 2013. It was discovered in *Neomys fodiens* captured between 2010 and 2012 in central Poland [33]. The unclassified Camp Ripley virus is the first hantavirus discovered in *Blarina brevicauda* captured in Minnesota in 1998 [32]. The unclassified Qiān Hú Shān virus was discovered in the lung tissue of stripe-backed shrews (*Sorex cylindricauda*) captured in 2005 in China [35]. The unclassified Xinyi virus was first discovered in Taiwanese mole shrews *(Anourosorex yamashinai*) captured in 1985 in Taiwan, China [30]. Phylogenetic analysis suggested that the Xinyi virus shares a common ancestor with the Cao Bằng virus. The unclassified Yákèshí virus was discovered in long-clawed shrews (*Sorex unguiculatus*) captured in 2006 in Yákèshí, Inner Mongolia. The unclassified Liánghé virus was found in *Anourosorex squamipes* captured between 2010 and 2011 in China [23].

Four species in the genus *Thottimvirus* of this family, such as *T. imjinense*, *T. thottapalayamense*, and two unclassified species, were detected in shrews (Table 2). Of them, *T. thottapalayamense* (Thottapalayam virus) was first isolated in 1964 in *Suncus murinus* in India. This virus represents the earliest isolation of a hantavirus in shrews [37]. *T. imjinense* (Imjin virus) was first discovered in *Crocidura lasiura* captured between 2004 and 2005 near the DMZ along the Imjin River in South Korea [36]. The unclassified Kilimanjaro virus and Uluguru virus in *Thottimvirus* were first discovered in Geata mouse shrews (*Myosorex geata*) and Kilimanjaro mouse shrews (*Myosorex zinki*) captured between 1995 and 2002 in Tanzania [39].

Except for Seoul virus, all the above hantanviruses were only found in shrews with unknown pathogenicity.

### 2.6. Nairovirid Viruses Detected in Shrews

The family *Nairoviridae* currently comprises seven genera and 51 species, which infect arthropods, birds, and mammals [61]. Several viruses in this family, such as the Crimean-Congo hemorrhagic fever virus and Nairobi sheep disease virus, can cause diseases in humans and/or domestic animals [61].

Six species in the genus *Orthonairovirus* of this family, namely *O. erveense*, *O. lambarenense*, *O. thiaforaense*, and three unclassified species, were detected in *Crocidura* spp. in multiple countries (Table 2). Of them, *O. erveense* (Erve virus) was first detected in 1982 from multiple organs of *Crocidura russula* trapped in Western France. Serological surveys indicated that Erve virus had a large apparent geographical distribution in France and infects rodents, insectivores, wild boars, red deer, sheep, herring gulls, and humans, leading to neurological symptoms in humans [40]. *O. lambarenense* (Lamusara virus) and the unclassified Lamgora virus in *Orthonairovirus* were discovered in *Crocidura* spp. captured between 2019 and 2020 in Gabon [41]. *O. thiaforaense* (Thiafora virus) was first discovered in *Crocidura* sp. captured in 1971 in Senegal [42].

Additionally, the unclassified Cencurut virus in *Orthonairovirus* was first discovered in *Suncus murinus* captured between 2012 and 2014 in Singapore [43]. An unclassified species of *Orthonairovirus* and an unclassified species of the genus *Xinspiovirus* of this family were detected in the lungs of Taiwanese gray shrews (*Crocidura tanakae*) [17]. These orthonairoviruses have been found only in shrews with unknown pathogenicity.

### 2.7. Othomyxovid Viruses Detected in Shrews

The family *Orthomyxoviridae* currently comprises nine genera and 21 species, which infect mammals and birds [62]. Several viruses in this family, such as influenza A virus and influenza B virus, can cause diseases in humans and/or domestic animals.

H5N6 subtype highly pathogenic avian influenza in the genus *Alphainfluenzavirus* of this family was detected in the lungs of *Anourosorex squamipes* [17]. This virus is highly pathogenic to chickens, mainly circulating in wild and domestic birds. It has sporadically infected some humans [63]. The shrew was likely accidentally infected with the avian virus.

### 2.8. Paramyxovid Viruses Detected in Shrews

The family *Paramyxoviridae* currently comprises four subfamilies, 17 genera, and 78 species [64]. Numerous viruses in this family, such as measles virus and canine distemper virus, cause diseases in humans or domestic animals.

Gamak virus and Daeryong virus in the genus *Henipavirus* were first reported in South Korea in recent years. They were both discovered in *Crocidura lasiura* and *Crocidura shantungensis*. These two viruses were exclusively detected in shrews. Gamak virus can infect and replicate in human hypotriploid alveolar basal epithelial cell lines and elicit the production of type I/III interferon, interferon-stimulated genes, and proinflammatory cytokines [44].

Twelve unclassified species in the genus *Henipavirus* of this family have been identified. Among them, Langya virus was first reported in China in 2022 with the reservoir hosts of shrews. The virus was detected in *Crocidura lasiura* and *Crocidura shantungensis*. The virus is pathogenic to humans, and 35 acute Langya virus cases manifested influenza-like symptoms. Previous research encompassed 25 wild animal species and indicated a high virus infection rate among the shrews (71/262, 27%) [45].

In *Henipavirus*, Melian virus was first detected in large-headed shrews (*Crocidura grandiceps*) captured in 2018 in Guinea [46]. Denwin virus was first detected in *Crocidura russula* captured in 2019 in Belgium [46]. Ninorex virus was first detected in *Sorex minutus* captured in 2020 in Belgium [47]. Phylogenetic analysis of these viruses and some other relevant paramyxoviruses suggested that both genera could be divided into two clades, one covering bat-borne viruses and the other covering rodent- and shrew-borne viruses [47].

Beilong virus in the genus *Jeilongvirus* of this family has been detected in rodents and shrews. A study in China showed a positivity rate of Beilong virus 28.57% (2/7) in the Asian lesser white-toothed shrews (*Crocidura shantungensis*) and 17.57% (13/74) in the *Suncus murinus* [48]. Currently, there is no evidence of human infection with Beilong virus, but its potential risk to humans and livestock should not be underestimated [65].

### 2.9. Phenuivirid Viruses Detected in Shrews

The family *Phenuiviridae* currently comprises 23 genera and 159 species, which infect vertebrates, including mammals and birds, invertebrates, plants, and fungi [66]. Some viruses in this family, such as sand-fly fever Naples virus and Chikungunya virus, can cause diseases in humans and/or domestic animals. Phenuivirid genomes consist of two or three single-stranded RNA molecules, some of which encode two proteins in non-overlapping ORFs of opposite polarities.

Severe fever with thrombocytopenia syndrome (SFTS) is an emerging hemorrhagic fever caused by SFTS virus (SFTSV), a species (*Dabie Bandavirus*) in the genus *Bandavirus* in *Phenuiviridae* [49]. SFTS has been reported in humans in China, South Korea, and Japan since 2010 [67]. The main clinical manifestations of SFTS include acute fever, thrombocytopenia, leukopenia, and gastrointestinal and neurological symptoms [6,7,8]. Moreover, multiple organ failure may occur in severe cases, with a maximum mortality rate of 30%. A survey suggested that shrews are more likely than other wild animals to be the reservoir of SFTSV because the prevalence of SFTSV in *Suncus murinus* is significantly higher than in other animals [49].

Diverse unclassified species in *Phenuiviridae* have been identified in *Anourosorex squamipes*, *Crocidura attenuata*, *and Chodsigoa smithii* in China in recent years [11]. It remains unknown whether these viruses were shrew viruses or the viruses of plants or fungi in shrew food.

### 2.10. Rhabdovirid Viruses Detected in Shrews

The family *Rhabdoviridae* currently comprises 56 genera that are assigned to four subfamilies and 434 species, which infect vertebrates, invertebrates, and plants [68]. A few rhabdoviruses, such as rabies virus and vesicular stomatitis virus, can cause diseases in humans and domestic animals.

Members of the genus *Lyssavirus* of this family infect a variety of mammals causing rabies-like diseases [69]. The species *L. mokola* was detected in Nigeria in 1968 from pooled lung, liver, spleen, kidney, and heart of shrews (*Crocidura* sp.) [69]. The species *L. rabies* (rabies virus) was identified in the lungs of *Suncus murinus* in China using HTS [17]. This finding is of public health significance, as it suggests that shrews could be involved in the ecology and epidemiology of rabies virus.

An unclassified species in the genus *Tupavirus* and eight unclassified species in unclassified genera of *Rhabdoviridae* were identified in shrews, such as *Chodsigoa smithii*, *Anourosorex squamipes*, *Crocidura attenuata*, *Blarinella griselda* (Indochinese short-tailed shrews), and *Anourosorex squamipes*, in China [11,17]. The pathogenicity of these shrew viruses remains unknown.

## 3. +ssRNA Viruses Detected in Shrews

Table 3 shows that 62 species of +ssRNA viruses in shrews have been detected.

### 3.1. Arterivirid Viruses Detected in Shrews

The family *Arteriviridae* currently comprises six subfamilies, 13 genera, and 23 species, which infect vertebrates, such as pigs, horses, and non-human primates [84]. Several significant veterinary pathogens, such as equine arteritis virus and porcine respiratory and reproduction syndrome virus 2, can cause severe diseases in domestic animals and/or non-human primates.

The genus *Muarterivirus* of *Arteriviridae* contains a shrew arterivirus, Olivier’s shrew virus 1, which was first detected in Olivier’s shrews *(Crocidura olivieri guineensis*) in 2016 in Guinea [70]. Additionally, six unclassified species of shrew arteriviruses were detected in *Crocidura shantungensis* and *Anourosorex squamipes* in China in recent years [11,17].

### 3.2. Astrovirid Viruses Detected in Shrews

The family *Astroviridae* currently comprises two genera and 22 species, which infect mammals (19 species) and birds (3 species). Some astroviruses, such as human astrovirus and duck astrovirus, can cause diseases in humans or domestic animals [85]. Infection with astroviruses often involves damage to livers, kidneys, or immune system. Duck astrovirus causes highly fatal hepatitis in ducklings [86].

Three unclassified species in the genus *Bastrovius*, three unclassified species in the genus *Mamastrovirus*, and one species in an unclassified genus of *Astroviridae* were detected in shrews, such as *Anourosorex squamipes*, *Crociduraattenuata*, *Chodsigoa smithii*, *Episoriculus leucops* (long-tailed brown-toothed shrews), and *Suncus murinus* in China in recent years.

### 3.3. Calicivirid Viruses Detected in Shrews

The family *Caliciviridae* currently comprises 11 genera, each including one or two species, which infect mammals, birds, or fish [87]. Some viruses in this family, such as norovirus and feline calicivirus, can cause diseases in humans or domestic animals.

Rabbit hemorrhagic disease virus in the genus *Lagovirus* of this family is highly pathogenic to rabbits. A survey identified this virus in a dead Mediterranean pine vole and two white-toothed shrews [72], which suggested that shrews could be victims of this virus.

### 3.4. Coronavirid Viruses Detected in Shrews

The family *Coronaviridae* currently comprises four subfamilies, six genera, and 54 species, which infect vertebrates (mammals, birds, amphibians, and fish) [88]. Some coronaviruses, such as severe acute respiratory syndrome-related coronavirus, infectious bronchitis virus, and porcine epidemic diarrhea virus, can cause diseases in humans or domestic animals.

Three unclassified shrew coronaviruses in the genus *Alphacoronavirus* of this family were detected in *Sorex araneus* and *Suncus murinus* captured in recent years in China [11,75]. Another unclassified shrew coronavirus in the genus *Alphacoronavirus* was detected in *Sorex araneus* captured in the United Kingdom between 2008 and 2015 [73]. These shrew viruses have been found only in shrews with unknown pathogenicity.

### 3.5. Flavivirid Viruses Detected in Shrews

The family *Flaviviridae* currently comprises four genera and 97 species, which infect various mammals [89,90]. Most members of the genus *Orthoflavivirus* are arthropod-borne. Some flaviviruses, such as yellow fever virus, Dengue virus, Zika virus, human hepatitis C virus, Japanese encephalitis virus, West Nile virus, tick-borne encephalitis virus, and Tambusu virus, can cause diseases in humans or domestic animals.

Tick-borne encephalitis virus (TBEV) in the genus *Orthoflavivirus* of this family can infect humans and cause meningitis, encephalitis, or meningoencephalitis. It circulates between ticks and small mammals. Hedgehogs, shrews, moles, and certain rodents are hosts of ticks and reservoirs of this virus. This virus was detected in 1967 in *Sorex araneus* in Slovakia [76].

Usutu virus in *Orthoflavivirus* is a Culex-associated mosquito-borne flavivirus that was also found in *Crocidura* sp. captured between 2012 and 2013 in Senegal. This virus was initially discovered in 1959 and is associated with mosquitoes as its vectors. Currently, the virus has been isolated from birds, arthropods, and humans in Europe and Africa, and subsequently detected in bats and horses [79].

Powassan virus 2 in *Orthoflavivirus* can lead to encephalitis in humans and is primarily transmitted by arthropod vectors. This virus has been reported not only in humans and other animals but also detected in *Blarina brevicauda* captured between 2018 and 2020 in Massachusetts and Rhode Island in the United States. Early studies suggested that groundhog ticks and squirrel ticks naturally maintain this virus with woodchucks and other medium-sized mammals, such as skunks and raccoons, or squirrels. These animals likely serve as the reservoir of the virus [78].

Shrews could be minor reservoirs or accidental hosts of the aforementioned TBEV, Usutu virus, and Powassan virus.

Rat pegivirus in *Pegivirus* was initially identified in the sera of desert woodrats in 2013, which was also discovered in *Suncus murinus* captured between 2013 and 2015 in China. This virus primarily targets lymphocytes and causes asymptomatic infections in humans and other animals [80].

An unclassified shrew flavivirus in the genus *Pegivirus*, three unclassified shrew flaviviruses in the genus *Hepacivirus*, and an unclassified shrew flavivirus in the genus *Pestivirus* were detected in *Suncus murinus*, *Chodsigoa smithii*, or *Crocidura attenuata* in China in recent years through HTS [11,80,91].

### 3.6. Hepevirid Viruses Detected in Shrews

The family *Hepeviridae* currently comprises six genera and 39 species, five genera and ten species, which infect mammals, birds, and fish [92]. Some viruses in this family, such as human hepatitis E virus and rabbit hepatitis E virus, can cause diseases in humans and/or domestic animals.

Three unclassified shrew hepevirus species were detected in *Crocidura attenuata*, *Chodsigoa smithii*, and *Crocidura attenuata* in China in recent years through HTS [11,17]. An unclassified shrew hepevirus species was detected in *Crocidura russula* in Germany in recent years through HTS as suggested by a sequence reported to GenBank.

### 3.7. Nodavirid Viruses Detected in Shrews

The family *Nodaviridae* currently comprises two genera, each including four or more species, which infect insects (*Alphanodavirus*) or fish (*Betanodavirus*) [93]. Some viruses in this family, such as the striped jack nervous necrosis virus, can cause diseases in animals.

Multiple unclassified species in this family with unknown pathogenicity were detected in *Anourosorex squamipes*, *Chodsigoa smithii*, *Crocidura attenuata*, and *Crocidura shantungensis* in China in recent years through HTS [11].

### 3.8. Picornavirid Viruses Detected in Shrews

The family *Picornaviridae* currently comprises 63 genera and 147 species which infect vertebrates (at least six of the seven classes) [94]. Numerous viruses in this family, such as poliovirus and foot-and-mouth disease virus, can cause diseases in humans or domestic animals.

An unclassified species in the genus of *Dicipivirus* of this family was detected in the lungs of *Crocidura lasiura* in China using HTS [17].

Hepatitis A virus (HAV) in the genus *Hepatovirus* of *Picornaviridae* is an ancient and ubiquitous human pathogen recovered previously only in primates. Human HAV could originate from the virus in rodents [82]. Although HAV-like viruses have been identified in *Sorex araneus* in Germany and in *Suncus murinus* in China, shrew HAVs are distinct from human HAV, and its pathogenicity to humans remains unknown [11,82]. Patterns of shrew HAV infection in shrews mimicked those of human HAV in hepatotropism, fecal shedding, acute nature, and extinction of the virus in a closed host population [74].

One unclassified species in the genus *Parechovirus* of *Picornaviridae* was detected in *Crocidura leucodon* and *Sorex antinorii* (Valais shrews) in Italy using RT-PCR [83]. Two unclassified species in the genus *Mischivirus*, one unclassified species in the genus *Parobovirus*, and eight species of unclassified genera in *Picornaviridae* from *Anourosorex squamipes*, *Blarinella griselda*, *Chodsigoa smithii*, *Crocidura attenuata*, *Crocidura lasiura*, *Sorex caecutiens*, and *Suncus murinus* in China were detected using the HTS [11,17]. The pathogenicity of these shrew viruses remains unknown.

## 4. dsDNA Viruses Detected in Shrews

Table 4 shows that seven species of dsDNA viruses in shrews have been detected.

### 4.1. Adenovirid Viruses Detected in Shrews

The family *Adenoviridae* currently comprises six genera and 109 species, which infect mammals, birds, reptiles, amphibians, and fish [100]. Some adenoviruses, such as human adenovirus 1 and canine adenovirus 1, can cause diseases in humans or domestic animals.

Two unclassified species with unknown pathogenicity in the genus *Mastadenovirus* of this family were detected in *Suncus murinus* in China and *Sylvisorex* sp. in Cameron, respectively, using PCR [95,96].

### 4.2. Hepadnavirid Viruses Detected in Shrews

The family *Hepadnaviridae* currently comprises five genera and 18 species, which infect mammals, birds, and fish [87]. Some viruses in this family, such as human hepatitis B virus and duck hepatitis B virus, can cause diseases in humans or domestic animals.

Shrew hepatitis B virus with unknown pathogenicity in the genus *Orthoepadnavirus* of this family has been detected in *Anourosorex squamipes*, *Crocidura attenuata*, and *Crocidura lasiura*, but not in *Suncus murinus*, captured in China in recent years [97]. Phylogenetic analysis revealed that shrew HBVs were closely related to a bat hepatitis B virus with a similar genome structure.

### 4.3. Orthoherpesvirid Viruses Detected in Shrews

The family *Orthoherpesviridae* currently comprises 17 genera and 118 species, which infect mammals, birds, and reptiles [101]. Some viruses in this family, such as Kaposi’s sarcoma-associated herpesvirus and pseudorabies virus, can cause diseases in humans or domestic animals.

Four shrew species with unknown pathogenicity in an unclassified genus of this subfamily were detected in *Crocidura* spp. in Cameroon and Congo [74].

### 4.4. Polyomavirid Viruses Detected in Shrews

The family *Polyomaviridae* currently comprises six genera and 112 species, which infect mammals, birds, and fish [102]. Some viruses in this family, such as JC virus and SV40, can cause diseases in humans or domestic animals.

An unclassified polyomavirus with unknown pathogenicity in *Sorex araneus* and *Sorex coronatus* (Millet’s shrews) was detected in Germany and Norway through PCR [98].

### 4.5. Poxvirid Viruses Detected in Shrews

The family *Poxviridae* currently comprises 22 genera and 83 species, which infect vertebrates and arthropods [103]. Some viruses in this family, such as variola virus and chicken poxvirus, can cause diseases in humans and/or domestic animals.

Monkeypox virus in the genus *Orthopoxvirus* of this family is zoonotic and has caused human infections in numerous countries in recent years. A survey in Zambia suggested that 14 of 42 shrews had antibodies against this virus [99]. However, the role of shrews in the epidemiology of this virus remains enigmatic. An unclassified species in this genus was detected in lung samples of Sorex *araneus* in Norway using PCR [54].

## 5. ssDNA Viruses Detected in Shrews

Table 5 shows that four species of ssDNA viruses in shrews have been detected.

### 5.1. Anellovirid Viruses Detected in Shrews

The family *Anelloviridae* currently comprises 34 genera and 173 species, which infect mammals, birds, and reptiles [108]. Some viruses in this family, such as Torque teno virus and Torque teno mini virus, are prevalent in humans and/or domestic animals. They could be associated with liver or respiratory diseases, hematological disorders, cancer, and immune suppression.

The species *Rhotorquevirus murid* 1 (Torque teno rodent virus) was detected in *Suncus murinus* captured in China in recent years through PCR [104].

### 5.2. Parvovirid Viruses Detected in Shrews

The family *Parvoviridae* currently comprises two subfamilies (*Parvovirinae* and *Densovirinae*), 13 genera, and > 75 species. Viruses in *Parvovirinae* infect vertebrates, including mammals, birds, and reptiles. Viruses in *Densovirinae* infect insects, crustacea, and echinoderms [109]. Some parvoviruses, such as parvovirus B19 and canine parvovirus, can cause diseases in humans or domestic animals.

The species *Rodent protoparvovirus* 1 (Mpulungu bufavirus) in the genus *Protoparvovirus* of this family was detected in lesser red musk shrews (*Crocidura hirta*) captured between 2011 and 2013 in Zambia using HTS and PCR [105].

A new genotype of porcine bocavirus in the genus *Bocaparvovirus* of *Parvoviridae* can cause respiratory and gastrointestinal diseases in pigs. This virus was detected in *Suncus murinus* captured between 2015 and 2017 in China, but shrews are unlikely its reservoir [106].

Adeno-associated virus in the genus *Dependoparvovirus* of *Parvoviridae* can infect humans and multiple domestic and wild animals, including pigs, cows, goats, chickens, snakes, bats, and rodents. It was detected in *Suncus murinus* captured between 2015 and 2017 in China [107].

## 6. dsRNA Viruses Detected in Shrews

Table 6 shows that 26 species of dsRNA viruses in shrews have been detected.

### 6.1. Picobirnavirid Viruses Detected in Shrews

The family *Picobirnaviridae* currently comprises one genus and three species, which infect vertebrates and invertebrates [112]. Some picobirnaviruses are widely distributed geographically among humans and other mammals. They have been mainly identified in fecal specimens and in raw sewage samples. The pathogenicity of picobirnaviruses has not been established. An unclassified picobirnavirus was identified in lungs of *Crocidura lasiura*, and *Crocidura shantungensis* in China using HTS [17].

### 6.2. Sedoreovirid Viruses Detected in Shrews

The family *Sedoreoviridae* currently comprises six genera and 39 species, which infect mammals, birds, crustaceans, arthropods, algae, and plants [113]. Some viruses in this family, such as rotavirus A, bluetongue virus, and African horse sickness virus, can cause diseases in humans or domestic animals.

Bluetongue virus in the genus *Orbivirus* of this family primarily infects sheep and various ruminant animals, with a high fatality rate in sheep. This virus can also infect rodents and carnivores and can be transmitted by certain mosquitoes. This virus was found in shrews in Africa in the 1990s [110]. The role of shrews in the epizootiology of bluetongue needs elucidation.

Rotavirus A in the genus *Rotavirus* of this family can infect humans and diverse domestic or wild animals. This virus and five unclassified species in this genus in *Anourosorex squamipes*, *Blarinella griselda*, *Chodsigoa smithii*, *Crocidura shantungensis*, *Crocidura attenuata*, and *Suncus murinus* captured in China in recent years were detected through HTS [11,111]. Additionally, three unclassified species of shrew sedoreoviruses in *Chodsigoa smithii* and *Crocidura attenuate* captured in China, which were distinct from known genera of this family, were detected in recent years through HTS [11,111].

### 6.3. Spinareovirid Viruses Detected in Shrews

The family *Spinareoviridae* currently comprises nine genera and 58 species, which infect mammals, aquatic animals (fish, mammals, crustaceans, and molluscs), birds, reptiles, arthropods, fungi, and plants [114]. Some spinareoviruses, such as human orthoreovirus and turkey orthoreovirus, can cause diseases in humans and domestic animals.

Two unclassified species in the genus *Cypovirus*, two unclassified species in the genus *Orthoreovirus*, and eight unclassified species distinct from known genera of this family were detected in *Chodsigoa smithii*, *Crocidura attenuata*, and *Suncus murinus* captured in China in recent years through HTS [11].

## 7. Discussion and Outlook

This review shows that shrew populations host at least 32 families and 190 species of viruses. Two recent publications regarding the large-scale detection of shrew viruses in China greatly expanded the knowledge about shrew virome or shrew viral diversity [11,17].

Dozens of other species of viruses have also been identified in shrews, but they were plant viruses or invertebrate viruses, and hence have not been described in this review [11,17]. They were detected in shrew samples, possibly due to their existence in shrew food or shrew surroundings.

Shrews have been identified with compelling evidence as the reservoirs of the zoonotic Langya virus, TBEV, SFTSV, and BDV-1. Shrews could be intermediate or incidental hosts or victims of Ebola virus, bluetongue virus, bufavirus, Usutu virus, tick-borne encephalitis virus, monkeypox virus, avian influenza virus, and RHDV, which are pathogenic to humans or domestic animals. Nevertheless, these infections have demonstrated that shrews are significant in the epidemiology and control of zoonosis and pose considerable threats to public health and animal health because shrews share overlapping habitats with humans and livestock. This highlights the surveys of shrew viruses for public health and animal welfare.

Dozens of virus species, such as Thottapalayam virus, Seewis virus, Thiafora virus, Wencheng shrew virus, *Suncus murinus* hepacivirus, and Olivier’s shrew virus 1, have been identified only in shrews. This suggests that shrews are likely the reservoirs of these species of viruses.

At least 24 species of hantaviruses, 13 species of paramyxoviruses, 17 species of phenuiviruses, 11 species of phabdoviruses, 9 species of flaviviruses, 13 species of nodaviruses, 13 species of nairoviruses, 11 species of sedoreoviruses, and 12 species of spinareoviruses in shrews have been identified (Figure 1). This suggests that shrews are likely the reservoirs of some species of these virus families, as it is unlikely that multiple species of the same virus family all occasionally infect shrews.

**Figure 2:**
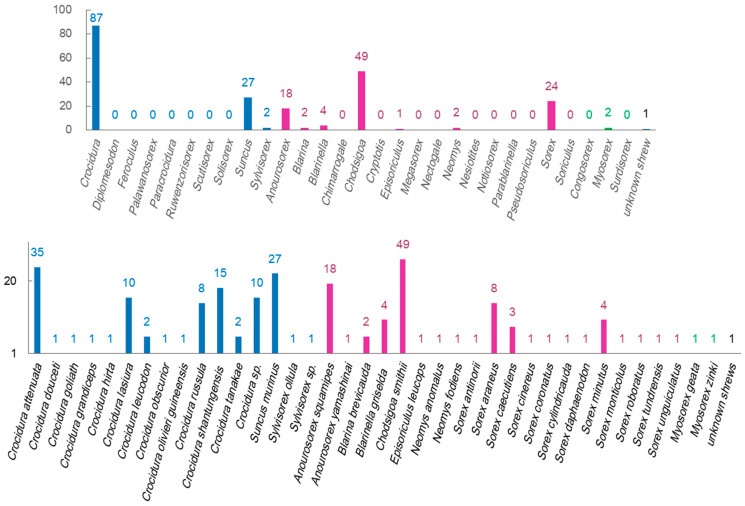
Numbers of virus species that have been identified in certain shrew genera and shrew species. The blue, purple, and green colors represent genera or species in the shrew subfamilies of *Crocidurinae*, *Soricinae*, and *Myosoricinae*, respectively.

The counts of identified virus species were significantly different among shrew genera and shrew species (Figure 2). The shrew genus *Crocidura* hosts more shrew species than other shrew genera, and the identified virus species were more from this shrew genus than from other shrew genera. *Chodsigoa smithii*, *Crocidura attenuate*, *Suncus murinus*, *Anourosorex squamipes*, *Crocidura shantungensis*, *Sorex araneus*, and *Crocidura lasiura* have been identified as being infected with more species of viruses than other shrew species. The factors, such as the abundance and distribution of these shrew genera or species, as well as the difficulty of capturing shrews of these genera or species, can affect the counts of identified virus species.

A database showed that 237, 201, and 204 species of viruses have been detected in *Mus musculus*, *Sus scrofa*, and *Bos taurus*, respectively [115]. With the consideration of the genetic diversity of shrews, most shrew viruses have yet to be identified so far. Meanwhile, much information regarding shrew viruses is based on viral sequences rather than viral isolates. The paucity of knowledge regarding virus diversity and viral isolates from shrews has hampered progress in demonstrating their pathogenic potential.

By virtue of their small size, voracious appetite, high metabolic rate, and aggressive behavior, most shrew species are difficult to handle and breed. However, it is desirable to develop efficient methods to breed them in laboratories because shrews are needed to investigate the transmission, pathogenicity, and potential control measures of shrew viruses, particularly those with biomedical significance [116].

Future research should assess the transmission risks of shrew viruses from shrews to humans and domestic animals and conduct broader surveys to delve into the diversity, evolution, and potential pathogenicity of these viruses. This will enhance our capacity to respond swiftly to the potential outbreaks of shrew viruses in humans or domestic animals with effective measures.

## Figures and Tables

**Table 1:** Geographic distribution and species number of 26 shrew genera.

Subfamily	Genus	Species	Geographical Distribution
*Crocidurinae*	*Crocidura*	222 species	Asia, Africa, and Europe
	*Diplomesodon*	1 species	Europe
	*Feroculus*	1 species	Asia
	*Palawanosorex*	1 species	Asia
	*Paracrocidura*	3 species	Africa
	*Ruwenzorisorex*	1 species	Africa
	*Scutisorex*	2 species	Africa
	*Solisorex*	1 species	Asia
	*Suncus*	20 species	Asia, Africa, Europe, and Oceania
	*Sylvisorex*	15 species	Africa
*Soricinae*	*Anourosorex*	4 species	Asia
	*Blarina*	5 species	North America
	*Blarinella*	2 species	Asia
	*Cryptotis*	54 species	North America and South America
	*Chimarrogale*	7 species	Asia
	*Chodsigoa*	10 species	Asia
	*Episoriculus*	8 species	Asia
	*Megasorex*	1 species	North America
	*Nectogale*	1 species	Asia
	*Nesiotites*	3 species	Asia
	*Neomys*	3 species	Europe and Asia
	*Notiosorex*	4 species	North America
	*Parablarinella*	2 species	Asia
	*Sorex*	86 species	Asia, Europe, North America and South America
	*Soriculus*	5 species	Asia
*Myosoricinae*	*Congosorex*	3 species	Africa
	*Myosorex*	19 species	Africa
	*Surdisorex*	3 species	Africa

**Table 2:** Detection of −ssRNA viruses in shrews using high-throughput sequencing (HTS), PCR, RT-PCR, serological methods, and/or virus isolation.

Virus Family and Genus	Virus Species and Name	Host Species	Country	Detection and References
*Arenaviridae*, *Mammarenavirus*	*Mammarenavirus wenzhouense* * (Wēnzhōu virus)	*Suncus murinus*	China	diverse samples_RT-PCR + isolation [12,13]; multiple organs and feces_HTS [11]
	*Mammarenavirus choriomeningitidis* * (lymphocytic choriomeningitis virus)	*Crocidura goliath*	Gabon	sera_serology [14]
	unclassified species (shrew mammarenavirus)	*Suncus murinus*	China	diverse samples_RT-PCR [15]
*Bornaviridae*, *Orthobornavirus*	*Orthobornavirus bornaense* * (Borna disease virus 1)	*Crocidura leucodon*	Switzerland; Germany	heart_RT-PCR [16]; multiple organs_HTS [OR713886] ^#^
*Chuviridae*, unclassified genera	unclassified species (Wufeng shrew chuvirus 1)	*Crocidura attenuata*; *Chodsigoa smithii*	China	multiple organs and feces_HTS [11]
	unclassified species (Wufeng shrew chuvirus 2)	*Chodsigoa smithii*	China	multiple organs and feces_HTS [11]
	unclassified species (Wufeng shrew chuvirus 5)	*Chodsigoa smithii*	China	multiple organs and feces_HTS [11]
	unclassified species (Wufeng shrew chuvirus 6)	*Crocidura attenuata*	China	multiple organs and feces_HTS [11]
	unclassified species (Crocidura lasiura chuvirus)	*Crocidura lasiura*	China	Lung_HTS [17]
*Filoviridae*, *Ebolavirus*	*Oblavirus percae* * (Ebola virus)	*Sylvisorex ollula*	Central African Republic	spleen_RT-PCR [18]
*Hantaviridae*, *Mobatvirus*	*Mobatvirus lenaense* (Lena virus)	*Sorex caecutiens*; *Sorex roboratus*	Russia	lung_RT-PCR [19]
*Hantaviridae*, *Orthohantavirus*	*Orthohantavirus artybashense* (Artybash virus)	*Sorex caecutiens*	Russia	lung_RT-PCR [20]
	*Orthohantavirus asikkalaense* (Asikkala virus)	*Sorex minutus*	Finland; the Czech Republic; Germany	diverse samples_RT-PCR [21]
	*Orthohantavirus boweense* (Bowé virus)	*Crocidura douceti*	Guinea	muscle_RT-PCR [22]
	*Orthohantavirus caobangense* (Cao Bằng virus; Wufeng shrew orthohantavirus 1; Wufeng shrew orthohantavirus 2)	*Anourosorex squamipes*; *Chodsigoa smithii*	Vietnam; China	diverse samples_RT-PCR [23]; multiple organs and feces_HTS [11]
	*Orthohantavirus jejuense* (Jeju virus)	*Crocidura shantungensis*	South Korea	lung + liver_RT-PCR [24]
	*Orthohantavirus kenkemeense* (Kenkeme virus)	*Sorex roboratus*	Russia	lung + liver_RT-PCR [25]
	*Orthohantavirus seoulense* * (Seoul virus)	*Crocidura lasiura*	China	lung_RT-PCR [26]
	*Orthohantavirus seewisense* (Seewis virus)	*Sorex araneus*; *Sorex tundrensis*; *Sorex daphaenodon*; *Sorex minutus*; *Neomys anomalus*	Switzerland, Germany, Slovakia, Hungary, Finland, Austria, Russia, Poland, and the Czech Republic	lung + liver_RT-PCR [27,28]
	unclassified species (Yákèshí virus)	*Sorex unguiculatus*	China	diverse samples_RT-PCR [23]
	unclassified species (Altai virus)	*Sorex araneus*	Russia	lung_RT-PCR + isolation [29]
	unclassified species (Xinyi virus)	*Anourosorex yamashinai*	China	multiple organs_RT-PCR [30]
	unclassified species (Ash River virus)	*Sorex cinereus*	United States	lung + liver_RT-PCR [31]
	unclassified species (Jemez Springs virus)	*Sorex monticolus*	United States	lung + liver_RT-PCR [31]
	unclassified species (Camp Ripley virus)	*Blarina brevicauda*	United States	lung + liver_RT-PCR [32]
	unclassified species (Boginia virus)	*Neomys fodiens*	Poland	lung_RT-PCR [33]
	unclassified species (Azagny virus)	*Crocidura obscurior*	Côte d’Ivoire	lung_RT-PCR [34]
	unclassified species (Qiān Hú Shān virus)	*Sorex cylindricauda*	China	lung_RT-PCR [35]
*Hantaviridae*, *Thottimvirus*	*Thottimvirus imjinense* (Imjin virus)	*Crocidura lasiura*	South Korea	diverse samples_RT-PCR + isolation [36]
	*Thottimvirus thottapalayamense* (Thottapalayam virus)	*Suncus murinus*	China	multiple organs and feces_HTS [11]
	*Thottimvirus thottapalayamense* (Thottapalayam virus)	*Suncus murinus*	India, Nepal	lung_RT-PCR + isolation [37,38]
	unclassified species (Kilimanjaro virus)	*Myosorex geata*	Tanzania	liver_RT-PCR [39]
	unclassified species (Uluguru virus)	*Myosorex zinki*	Tanzania	liver_RT-PCR [39]
*Nairoviridae*, *Orthonairovirus*	Orthonairovirus erveense * (Erve virus)	*Crocidura russula*	France, Germany	multiple organs_RT-PCR [40] [OR713866] ^#^
	*Orthonairovirus lambarenense* (Lamusara virus)	*Crocidura* sp.	Gabon	kidney_RT-PCR [41]
	*Orthonairovirus thiaforaense* (Thiafora virus)	*Crocidura* sp.	Senegal	multiple organs_HTS [42]
	unclassified species (Rasenna virus)	*Crocidura russula*	Germany	multiple organs_HTS [OR713845] ^#^
	unclassified species (Lamgora virus)	*Crocidura* sp.	Gabon	kidney_RT-PCR [41]
	unclassified species (Cencurut virus)	*Suncus murinus*	Singapore	Lung + spleen + kidney_HTS [43]
	unclassified species (*Crocidura tanakae* nairovirus 1)	*Crocidura tanakae*	China	Lung_HTS [17]
*Nairoviridae*, *Xinspiovirus*	unclassified species (*Crocidura tanakae* nairovirus 1)	*Crocidura tanakae*	China	Lung_HTS [17]
*Orthomyxoviridae*, *Alphainfluenzavirus*	*Alphainfluenzavirus influenzae* (influenza A virus)	*Anourosorex squamipes*	China	Lung_HTS [17]
*Paramyxoviridae*, *Henipavirus*	unclassified species (Daeryong virus)	*Crocidura lasiura*; *Crocidura shantungensis*	South Korea	kidney_HTS [44]
	unclassified species (Langya virus)	*Crocidura lasiura*; *Crocidura shantungensis*	China	diverse samples_HTS + isolation [45]
	unclassified species (Melian virus)	*Crocidura grandiceps*	Guinea	kidney_HTS [46]
	unclassified species (Denwin virus)	*Crocidura russula*	Belgium; Germany	kidney_HTS [46] multiple organs_HTS; [OR713882] ^#^
	unclassified species (Hasua virus)	*Crocidura russula*	Germany	multiple organs_HTS [OR713881] ^#^
	unclassified species (Lechcodon virus)	*Crocidura russula*	Germany	multiple organs_HTS [OR713879] ^#^
	unclassified species (Resua virus)	*Crocidura russula*	Germany	multiple organs_HTS [OR713876] ^#^
	unclassified species (Ninorex virus)	*Sorex minutus*	Belgium	kidney_HTS [47]
	unclassified species (Jinmen shrew henipavirus 1)	*Crocidura shantungensis*; *Crocidura attenuata*	China	multiple organs and feces_HTS [11]
	unclassified species (Jinmen shrew henipavirus 2)	*Crocidura shantungensis*	China	multiple organs and feces_HTS [11]
	unclassified species (Wenzhou shrew henipavirus 1)	*Suncus murinus*	China	multiple organs and feces_HTS [11]
	unclassified species (Wenzhou shrew henipavirus 2)	*Chodsigoa smithii*	China	multiple organs and feces_HTS [11]
*Paramyxoviridae*, *Jeilongvirus*	*Jeilongvirus beilongi* (Beilong virus)	*Crocidura shantungensis*; *Suncus murinus*	China	diverse samples_RT-PCR [48]
*Phenuiviridae*, *Bandavirus*	*Bandavirus dabieense* * (severe fever with thrombocytopenia syndrome virus)	*Suncus murinus*	China	serum + spleen_RT-PCR + serology [49]
*Phenuiviridae*, *unclassified genera*	unclassified species (Wufeng shrew phenuivirus 1)	*Anourosorex squamipes*	China	multiple organs and feces_HTS [11]
	unclassified species (Wufeng shrew phenuivirus 2)	*Crocidura attenuata*	China	multiple organs and feces_HTS [11]
	unclassified species (Wufeng shrew phenuivirus 3)	*Chodsigoa smithii*	China	multiple organs and feces_HTS [11]
	unclassified species (Wufeng shrew phenuivirus 4)	*Chodsigoa smithii*	China	multiple organs and feces_HTS [11]
	unclassified species (Wufeng shrew phenuivirus 5)	*Crocidura attenuata*	China	multiple organs and feces_HTS [11]
	unclassified species (Wufeng shrew phenuivirus 6)	*Crocidura attenuata*	China	multiple organs and feces_HTS [11]
	unclassified species (Wufeng shrew phenuivirus 7)	*Anourosorex squamipes*	China	multiple organs and feces_HTS [11]
	unclassified species (Wufeng shrew phenuivirus 8)	*Crocidura attenuata*	China	multiple organs and feces_HTS [11]
	unclassified species (Wufeng shrew phenuivirus 9)	*Anourosorex squamipes*	China	multiple organs and feces_HTS [11]
	unclassified species (Wufeng shrew phenuivirus 10)	*Anourosorex squamipes*	China	multiple organs and feces_HTS [11]
	unclassified species (Wufeng shrew phenuivirus 11)	*Chodsigoa smithii*	China	multiple organs and feces_HTS [11]
	unclassified species (Wufeng shrew phenuivirus 12)	*Chodsigoa smithii*	China	multiple organs and feces_HTS [11]
	unclassified species (Wufeng shrew phenuivirus 13)	*Chodsigoa smithii*	China	multiple organs and feces_HTS [11]
	unclassified species (Wufeng shrew phenuivirus 14)	*Chodsigoa smithii*	China	multiple organs and feces_HTS [11]
	unclassified species (Wufeng shrew phenuivirus 15)	*Crocidura attenuata*	China	multiple organs and feces_HTS [11]
	unclassified species (Wufeng shrew phenuivirus 16)	*Chodsigoa smithii*	China	multiple organs and feces_HTS [11]
*Rhabdoviridae*, *Lyssavirus*	*Lyssavirus mokola* (Mokola virus)	*Crocidura* sp.	Nigeria	multiple organs_isolation + serology + sequencing [50]
	*Lyssavirus rabies* (rabies virus)	*Suncus murinus*	China	Lung_HTS [17]
*Rhabdoviridae*, *Tupavirus*	unclassified species (Wufeng shrew tupavirus 1)	*Chodsigoa smithii*	China	multiple organs and feces_HTS [11]
*Rhabdoviridae*, *unclassified genera*	unclassified species (Wufeng shrew rhabdovirus 2)	*Anourosorex squamipes*; *Chodsigoa smithii*	China	multiple organs and feces_HTS [11]
	unclassified species (Wufeng shrew rhabdovirus 4)	*Anourosorex squamipes*	China	multiple organs and feces_HTS [11]
	unclassified species (Wufeng shrew rhabdovirus 6)	*Crocidura attenuata*	China	multiple organs and feces_HTS [11]
	unclassified species (Wufeng shrew rhabdovirus 7)	*Chodsigoa smithii*	China	multiple organs and feces_HTS [11]
	unclassified species (Wufeng shrew rhabdovirus 10)	*Chodsigoa smithii*	China	multiple organs and feces_HTS [11]
	unclassified species (Wufeng shrew rhabdovirus 15)	*Blarinella griselda*	China	multiple organs and feces_HTS [11]
	unclassified species (Wufeng shrew rhabdovirus 18)	*Anourosorex squamipes*	China	multiple organs and feces_HTS [11]
	unclassified species (*Anourosorex squamipes* rhabdovirus)	*Anourosorex squamipes*	China	Lung_HTS [17]
*Bunyavirales*, unclassified families	unclassified species (Wufeng shrew bunyavirus 1)	*Crocidura attenuata*	China	multiple organs and feces_HTS [11]
	unclassified species (Wufeng shrew bunyavirus 2)	*Anourosorex squamipes*	China	multiple organs and feces_HTS [11]
	unclassified species (Wufeng shrew bunyavirus 3)	*Chodsigoa smithii*	China	multiple organs and feces_HTS [11]
	(Wufeng shrew bunyavirus 4)	*Crocidura attenuata*	China	multiple organs and feces_HTS [11]

Note: in the tables of this review, those viruses with known pathogenicity to humans or domestic animals are marked with asterisks (*); those shrew viruses supported by sequences rather than by reports were given the GenBank accession numbers of the relevant sequences and marked with hashtags (#).

**Table 3:** Detection of +ssRNA viruses in shrews using high-throughput sequencing (HTS), PCR, RT-PCR, serological methods, and/or virus isolation.

Virus Family and Genus	Virus Species and Name	Host Species	Country	Detection and References
*Arteriviridae*, *Muarterivirus*	*Muarterivirus afrigant* (Olivier’s shrew virus 1)	*Crocidura olivieri guineensis*	Guinea	diverse sample_HTS [70]
	unclassified species (Wufeng shrew arterivirus 1)	*Anourosorex squamipes*	China	multiple organs_HTS [11]
	unclassified species (Jingmen shrew arterivirus 1)	*Crocidura shantungensis*	China	multiple organs_HTS [11]
	unclassified species (Jingmen shrew arterivirus 2)	*Crocidura shantungensis*	China	multiple organs_HTS [11]
*Arteriviridae*, unclassified genus	unclassified species (*Crocidura shantungensis* arterivirus 1)	*Crocidura shantungensis*	China	Lung_HTS [17]
	unclassified species (*Crocidura shantungensis* arterivirus 2)	*Crocidura shantungensis*	China	Lung_HTS [17]
	unclassified species (*Crocidura shantungensis* arterivirus 3)	*Crocidura shantungensis*	China	Lung_HTS [17]
*Astroviridae*, *Bastrovius*	unclassified species (Wufeng shrew bastrovirus 1)	*Chodsigoa smithii*	China	multiple organs and feces_HTS [11]
*Astroviridae*, *Bastrovius*	unclassified species (Wufeng shrew bastrovirus 2)	*Chodsigoa smithii*	China	multiple organs and feces_HTS [11]
*Astroviridae*, *Bastrovius*	unclassified species (Wufeng shrew bastrovirus 3)	*Chodsigoa smithii*	China	multiple organs and feces_HTS [11]
*Astroviridae*, *Mamastrovirus*	unclassified species (shrew astrovirus)	*Episoriculus leucops*; *Crociduraattenuata*	China	feces_RT-PCR [71]
*Astroviridae*, *Mamastrovirus*	unclassified species (Jingmen_shrew_astrovirus 1)	*Crocidura shantungensis*	China	multiple organs and feces_HTS [11]
*Astroviridae*, *Mamastrovirus*	unclassified species (Wufeng shrew_astrovirus 1)	*Anourosorex squamipes*	China	multiple organs and feces_HTS [11]
*Astroviridae*, unclassified genera	unclassified species (Wenzhou rodent astrovirus 1)	*Suncus murinus*	China	multiple organs and feces_HTS [11]
*Caliciviridae*, *Lagovirus*	*Lagovirus europaeus* * (rabbit hemorrhagic disease virus)	*Crocidura russula*	Spain	liver_RT-PCR [72]
*Coronaviridae*, *Alphacoronavirus*	unclassified species (shrew alphacoronavirus)	*Sorex araneus*	United Kingdom	liver_RT-PCR [73]
	unclassified species (Wencheng shrew coronavirus)	*Suncus murinus*	China	rectum_RT-PCR [74]
	unclassified species (Common shrew coronavirus)	*Sorex araneus*	China	pharyngeal and anal swabs_HTS [75]
	unclassified species (Wufeng shrew alphacoronavirus 1)	*Suncus murinus*	China	multiple organs and feces_HTS [11]
*Flaviviridae*, *Orthoflavivirus*	*Orthoflavivirus encephalitidis* * (tick-borne encephalitis virus)	*Sorex araneus*	Slovakia	sera_RT-PCR + isolation [76,77]
	*Orthoflavivirus powassanense* * (Powassan virus 2)	*Blarina brevicauda*	United States	spleen + brain_PCR [78]
	*Orthoflavivirus usutuense* * (Usutu virus)	*Crocidura* sp.	Senegal	brain_RT-PCR + isolation [79]
*Flaviviridae*, *Pegivirus*	*Pegivirus neotomae* * (rodent pegivirus)	*Suncus murinus*	China	sera_RT-PCR [80]
*Flaviviridae*, *Pegivirus*	unclassified species (Wufeng shrew pegivirus 2)	*Chodsigoa smithii*	China	multiple organs and feces_HTS [11]
*Flaviviridae*, *Hepacivirus*	unclassified species (Suncus murinus hepacivirus)	*Suncus murinus*	China	multiple organs and feces_HTS [11]
*Flaviviridae*, *Hepacivirus*	unclassified species (Wufeng rodent hepacivirus 3)	*Chodsigoa smithii*	China	multiple organs and feces_HTS [11]
*Flaviviridae*, *Hepacivirus*	unclassified species (Wufeng rodent hepacivirus 3)	*Chodsigoa smithii*	China	multiple organs and feces_HTS [11]
*Flaviviridae*, *Hepacivirus*	unclassified species (Wufeng shrew hepacivirus 1)	*Crocidura attenuata*	China	multiple organs and feces_HTS [11]
*Flaviviridae*, *Pestivirus*	unclassified species (Wufeng shrew pestivirus 1)	*Chodsigoa smithii*	China	multiple organs and feces_HTS [11]
*Hepeviridae*, *Paslahepevirus*	unclassified species (shrew hepatitis E virus)	*Suncus murinus*	China	serum_RT-PCR + serology [81]
*Hepeviridae*, unclassified genera	unclassified species (Wufeng shrew hepevirus 2)	*Crocidura attenuata*; *Chodsigoa smithii*	China	multiple organs and feces_HTS [11]
	unclassified species (Wufeng shrew hepe-like virus 1)	*Chodsigoa smithii*; *Crocidura attenuata*	China	multiple organs and feces_HTS [11]
	unclassified species (Wufeng shrew hepevirus_2)	*Chodsigoa smithii*; *Crocidura attenuata*	China	multiple organs and feces_HTS [11]
	unclassified species (shrew hepatitis E virus)	*Crocidura russula*	Germany	multiple organs_HTS [OR713884] ^#^
	unclassified species (Crocidura lasiura hepevirus)	*Crocidura lasiura*	China	Lung_HTS [17]
*Nodaviridae*, unclassified genus	unclassified species (Jingmen shrew nodavirus 1)	*Crocidura shantungensis*	China	multiple organs and feces_HTS [11]
	unclassified species (Wufeng shrew nodavirus 1)	*Chodsigoa smithii*	China	multiple organs and feces_HTS [11]
	unclassified species (Wufeng shrew nodavirus 2)	*Chodsigoa smithii*; *Crocidura attenuata*	China	multiple organs and feces_HTS [11]
	unclassified species (Wufeng shrew nodavirus 4)	*Chodsigoa smithii*	China	multiple organs and feces_HTS [11]
	unclassified species (Wufeng shrew nodavirus 6)	*Chodsigoa smithii*	China	multiple organs and feces_HTS [11]
	unclassified species (Wufeng shrew nodavirus 7)	*Crocidura attenuata*; *Chodsigoa smithii*	China	multiple organs and feces_HTS [11]
	unclassified species (Wufeng shrew nodavirus 8)	*Crocidura attenuata*; *Anourosorex squamipes*	China	multiple organs and feces_HTS [11]
	unclassified species (Wufeng shrew nodavirus 10)	*Crocidura attenuata*	China	multiple organs and feces_HTS [11]
	unclassified species (Wufeng shrew nodavirus 12)	*Anourosorex squamipes*	China	multiple organs and feces_HTS [11]
	unclassified species (Wufeng shrew nodavirus 13)	*Crocidura attenuata*; *Blarinella griselda*	China	multiple organs and feces_HTS [11]
	unclassified species (Wufeng shrew nodavirus 15)	*Chodsigoa smithii*	China	multiple organs and feces_HTS [11]
	unclassified species (Wufeng shrew nodavirus 16)	*Chodsigoa smithii*	China	multiple organs and feces_HTS [11]
	unclassified species (Wufeng shrew nodavirus 17)	*Crocidura attenuata*	China	multiple organs and feces_HTS [11]
*Picornaviridae*, *Dicipivirus*	unclassified species (*Crocidura lasiura* picornavirus 2)	*Crocidura lasiura*	China	Lung_HTS [17]
*Picornaviridae*, *Hepatovirus*	*Hepatovirus ishrewi* (shrew hepatovirus)	*Sorex araneus*	Germany	diverse samples_RT-PCR + isolation [82]
	unclassified species (Wufeng shrew_picornavirus 1)	*Chodsigoa smithii*	China	multiple organs and feces_HTS [11]
*Picornaviridae*, *Mischivirus*	*Mischivirus ehoushre* (Mischivirus E)	*Suncus murinus*	China	multiple organs and feces_HTS [11]
	unclassified species (Wufeng shrew picornavirus 3)	*Crocidura attenuata*	China	multiple organs and feces_HTS [11]
*Picornaviridae*, *Parechovirus*	*Parechovirus beljungani* * (Ljungan virus)	*Crocidura leucodon*; *Sorex antinorii*	Italy	multiple organs_RT-PCR [83]
*Picornaviridae*, *Parabovirus*	unclassified species (*Sorex caecutiens* picornavirus)	*Sorex caecutiens*	China	Lung_HTS [17]
*Picornaviridae*, unclassified genera	unclassified species (Wufeng shrew picornavirus 4)	*Anourosorex squamipes*	China	multiple organs and feces_HTS [11]
	unclassified species (Wenzhou shrew picornavirus 1)	*Suncus murinus*; *Chodsigoa smithii*; *Crocidura attenuata*	China	multiple organs and feces_HTS [11]
	unclassified species (Wufeng shrew picornavirus 2)	*Chodsigoa smithii*	China	multiple organs and feces_HTS [11]
	unclassified species (Wufeng shrew_picornavirus 5)	*Chodsigoa smithii*	China	multiple organs and feces_HTS [11]
	unclassified species (Wufeng shrew picornavirus 6)	*Blarinella griselda*	China	multiple organs and feces_HTS [11]
	unclassified species (*Crocidura lasiura* picornavirus 1)	*Crocidura lasiura*	China	Lung_HTS [17]
	unclassified species (*Suncus murinus* picornavirus 12)	*Suncus murinus*	China	Lung_HTS [17]
	unclassified species (*Suncus murinus* picornavirus 294)	*Suncus murinus*	China	Lung_HTS [17]
*Hepelivirales* unclassified family	unclassified species (Wufeng shrew hepe-like virus 1)	*Chodsigoa smithii*; *Crocidura attenuata*	China	multiple organs and feces_HTS [11]
*Nodamuvirales* unclassified family	unclassified species (Wufeng shrew nodamuvirus 1)	*Crocidura attenuata*	China	multiple organs and feces_HTS [11]
*Nodamuvirales* unclassified family	unclassified species (Wufeng shrew nodamuvirus 2)	*Crocidura attenuata*	China	multiple organs and feces_HTS [11]

Note: * and #, see the note of Table 2.

**Table 4:** Detection of dsDNA viruses in shrews using high-throughput sequencing (HTS), PCR, RT-PCR, serological methods, and/or virus isolation.

Virus Family and Genus	Virus Species and Name	Host Species	Country	Detection and References
*Adenoviridae*, *Mastadenovirus*	unclassified species (Asian house shrew adenovirus)	*Suncus murinus*; *Sorex araneus*	China	feces_PCR [95]
	unclassified species (shrew adenovirus)	*Sylvisorex* sp.	Cameroon	feces_PCR [96]
*Hepadnaviridae*, *Orthohepadnavirus*	*Orthohepadnavirus soricisinensis* (shrew hepatitis B virus)	*Anourosorex squamipes*; *Crocidura attenuata*; *Crocidura lasiura*	China	liver_PCR [97]
*Orthoherpesviridae*, unclassified genus	four unclassified species (shrew orthoherpesvirus)	*Crocidura* spp.	Cameroon and Congo	liver + spleen_PCR [74]
*Polyomaviridae*, *Alphapolyomavirus*	unclassified *Alphapolyomavirus* * (human polyomavirus 12)	*Sorex araneus*; *Sorex coronatus*; *Sorex minutus*	Germany and Norway	diverse samples_PCR [98]
*Poxviridae*, *Orthopoxvirus*	*Orthopoxvirus monkeypox* * (monkeypox virus)	*Crocidura* spp.	Zambia and Congo	sera + spleen_PCR + serology [99]
	unclassified species (shrew orthopoxvirus)	*Sorex araneus*	Norway	lung_PCR [54]

Note: *, see the note of Table 2.

**Table 5:** Detection of ssDNA viruses in shrews using high-throughput sequencing (HTS), PCR, RT-PCR, serological methods, and/or virus isolation.

Virus Family and Genus	Virus Species and Name	Host Species	Country	Detection and References
*Anelloviridae*, *Rhotorquevirus*	*Rhotorquevirus murid* 1 (Torque teno rodent virus)	*Suncus murinus*	China	throat + sera_PCR [104]
*Parvoviridae*, *Protoparvovirus*	*Rodent protoparvovirus* 1 (Mpulungu bufavirus)	*Crocidura hirta*	Zambia	spleen + intestinal contents_HTS+ PCR [105]
*Parvoviridae*, *Bocaparvovirus*	unclassified species * (porcine bocavirus G4)	*Suncus murinus*	China	feces + sera_PCR [106]
*Parvoviridae*, *Dependoparvovirus*	unclassified species (shrew adeno-associated virus)	*Suncus murinus*	China	feces + sera_PCR [107]

Note: *, see the note of Table 2.

**Table 6:** Detection of dsRNA viruses in shrews using high-throughput sequencing (HTS), PCR, RT-PCR, serological methods, and/or virus isolation.

Virus Family and Genus	Virus Species and Name	Host Species	Country	Detection and References
*Picobirnaviridae unclassified genus*	unclassified species (shrew picobirnavirus 1)	*Crocidura lasiura*; *Crocidura shantungensis*	China	Lung_HTS [17]
*Sedoreoviridae*, *Orbivirus*	*Orbivirus caerulinguae* * (Bluetongue virus)	*unknown shrews*	Unknown (Africa)	sera_serology [110]
	unclassified species (Wufeng shrew orbivirus)	*Chodsigoa smithii*	China	multiple organs and feces_HTS [11]
*Sedoreoviridae*, *Rotavirus*	*Rotavirus alphagastroenteritidis* * (rotavirus A)	*Suncus murinus*; *Chodsigoa smithii*	China	feces_RT-PCR [111]; multiple organs and feces_HTS [11]
	unclassified species (Jingmen_shrew rotavirus_1)	*Crocidura shantungensis*	China	multiple organs and feces_HTS [11]
	unclassified species (Wufeng shrew_rotavirus 1)	*Blarinella griselda*; *Crocidura attenuata*	China	multiple organs and feces_HTS [11]
	unclassified species (Wufeng shrew_rotavirus 2)	*Anourosorex squamipes*; *Chodsigoa smithii*	China	multiple organs and feces_HTS [11]
	unclassified species (Crocidura shantungensis rotavirus 9)	*Crocidura shantungensis*	China	Lung_HTS [17]
	(Crocidura shantungensis rotavirus 10)	*Crocidura shantungensis*	China	Lung_HTS [17]
*Sedoreoviridae*, unclassified genera	unclassified species (Wufeng shrew reo-like virus 2)	*Crocidura attenuata*	China	multiple organs and feces_HTS [11]
	unclassified species (Wufeng shrew reo-like virus 8)	*Chodsigoa smithii*	China	multiple organs and feces_HTS [11]
	unclassified species (Wufeng shrew reo-like virus 3)	*Crocidura attenuata*	China	multiple organs and feces_HTS [11]
*Spinareoviridae*, *Cypovirus*	unclassified species (Wufeng shrew cypovirus 1)	*Crocidura attenuata*	China	multiple organs and feces_HTS [11]
	unclassified species (Wufeng shrew cypovirus 2)	*Crocidura attenuata*	China	multiple organs and feces_HTS [11]
*Spinareoviridae*, *Orthoreovirus*	unclassified species (Wenzhou shrew orthoreovirus 1)	*Suncus murinus*	China	multiple organs and feces_HTS [11]
	unclassified species (mammalian orthoreovirus)	*Crocidura shantungensis*	China	multiple organs and feces_HTS [11]
*Spinareoviridae*, unclassified genera	unclassified species (Wufeng shrew reovirus 1)	*Chodsigoa smithii*	China	multiple organs and feces_HTS [11]
	unclassified species (Wenzhou shrew reovirus 1)	*Suncus murinus*	China	multiple organs and feces_HTS [11]
	unclassified species (Wenzhou shrew reovirus 2)	*Suncus murinus*	China	multiple organs and feces_HTS [11]
	unclassified species (Wufeng shrew reo-like virus 1)	*Chodsigoa smithii*; *Crocidura attenuata*	China	multiple organs and feces_HTS [11]
	unclassified species (Wufeng shrew reo-like virus 4)	*Chodsigoa smithii*	China	multiple organs and feces_HTS [11]
	unclassified species (Wufeng shrew reo-like virus 6)	*Chodsigoa smithii*	China	multiple organs and feces_HTS [11]
	unclassified species (Wufeng shrew reo-like virus 7)	*Chodsigoa smithii*	China	multiple organs and feces_HTS [11]
	unclassified species (Wufeng shrew reo-like virus 10)	*Crocidura attenuata*	China	multiple organs and feces_HTS [11]
*Reovirales*, unclassified family	unclassified species (Wufeng shrew reo-like virus 5)	*Chodsigoa smithii*	China	multiple organs and feces_HTS [11]
	unclassified species (Wufeng shrew_reo-like_virus_9)	*Chodsigoa smithii*	China	multiple organs and feces_HTS [11]

Note: * and #, see the note of Table 2.

## Data Availability

The data that support the findings of this study are available from the corresponding author Ji-Ming Chen upon reasonable request.

## Supplementary Materials

The following supporting information can be downloaded at: https://www.mdpi.com/article/10.3390/v16091441/s1, Table S1. Detailed information regarding the viruses having been identified in shrews.
